# Circulating long-chain n-3 polyunsaturated fatty acid and incidence of stroke: a meta-analysis of prospective cohort studies

**DOI:** 10.18632/oncotarget.19530

**Published:** 2017-07-25

**Authors:** Bo Yang, Xiao-Li Ren, Hong Huang, Xiao-Juan Guo, Ai-Guo Ma, Duo Li

**Affiliations:** ^1^ School of Public Health, Qingdao University, Qingdao, China; ^2^ Key Laboratory of Watershed Science and Health of Zhejiang Province, School of Public Health, Wenzhou Medical University, Wenzhou, China; ^3^ The Laboratory of Animal Center, Wenzhou Medical University, Wenzhou, China

**Keywords:** biomarker, circulation, meta-analysis, PUFA, stroke

## Abstract

**Background:**

Circulating long-chain (LC) n-3 polyunsaturated fatty acid (PUFA) can provide objective measures that reflect both dietary consumption and relevant biological processes. Nevertheless, prospective cohort studies on circulating LC n-3 PUFA in relation to incidence of stroke have yielded inconsistent results. We therefore conducted a meta-analysis to quantitatively evaluate the association.

**Results:**

A total of 2,836 stroke events occurred among 20,460 individuals aged 35–79 yr from 10 prospective cohort studies. Circulating LC n-3 PUFA was significantly associated with reduced risk of stroke (RR: 0.86; 95% CI: 0.76, 0.98; *I*^2^ = 0.00%), especially 22:5n-3 (RR: 0.74; 95% CI: 0.60, 0.92) and 22:6n-3 (RR: 0.78; 95% CI: 0.65, 0.94). The associations were more pronounced with ischemic stroke (RR: 0.81; 95% CI: 0.68, 0.96), but not with hemorrhagic stroke (RR: 0.95; 95% CI: 0.60, 1.49). A 1% increment of 22:5n-3 and 22:6n-3 proportions in circulating blood was associated with 25% (RR: 0.75; 95% CI: 0.64, 0.87) and 11% (RR: 0.89; 95% CI: 0.83, 0.95) reduced risk of stroke, respectively.

**Materials and Methods:**

Pertinent studies were identified from Cochrane Library, PubMed and EMBASE database through June 2017. Multivariate-adjusted risk ratios (RRs) with 95% confidence interval (CI) for incident stroke when comparing the top with the bottom tertiles of baseline LC n-3 PUFA proportions in blood were pooled using a random-effect model.

**Conclusions:**

Circulating LC n-3 PUFAs were linearly associated with reduced risk of stroke, especially 22:5n-3 and 22:6n-3. Such findings highlight the importance of circulating LC n-3 PUFA in the development of ischemic stroke.

## INTRODUCTION

Stroke can traditionally be classified into two major categories: ischemic stroke (IS) and hemorrhagic stroke (HS). Accumulating data have shown that stroke is the leading cause of acquired disability and disease burden in adults in most developed and developing regions, with heavy economic and social costs owing to functional impairments [1]. Thus, the primary prevention for stroke has always been an important public health priority.

Long-chain (LC) n-3 polyunsaturated fatty acid (PUFA), mainly 20:5n-3 (eicosapentaenoic acid, EPA), 22:5n-3 (docosapentaenoic acid, DPA) and 22:6n-3 (docosahexaenoic acid, DHA), which is principally derived from fish or seafood consumption, has been associated with reduced blood pressure and blood triglyceride levels. Two prior meta-analyses on association between LC n-3 PUFA intake and stroke have been published, with one meta-analysis revealing a remarkably negative association [2], but another meta-analysis reporting no statistically significant association [3]. Each study included in the two meta-analyses used dietary questionnaires to estimate LC n-3 PUFA intake, which could potentially lead to dietary measurement errors or bias. Moreover, individuals with high-risk of stroke who changed their dietary habit after the diagnosis may not have been excluded from study populations, which may bias benefit for stroke towards null.

In contrast to questionnaire estimates, circulating levels of LC n-3 PUFA can provide objective biomarkers of exposure that reflect both dietary consumption and biologically relevant processes, and also permits direct evaluation of individual LC n-3 PUFA that may have different effects on certain biological pathways or clinical end points. Convincing evidence from extensive experiments has demonstrated that the incorporation of LC n-3 PUFA into membrane phospholipids can modulate mechanisms of development and progression of stroke [4], possibly through increasing biomembrane fluidity [5], diminishing formation of free radicals [6], and inhibiting apoptotic pathways [7]. To date, whether circulating LC n-3 PUFA as a biomarker was associated with risk of stroke and its subtypes remains unclear presently, as most prospective cohort studies found no significant associations and only a few reported significantly inverse associations. Nevertheless, the possibility that a weak association may have been missed because of lower statistical power that cannot be excluded. We therefore conducted a meta-analysis to investigate the relationship between circulating LC n-3 PUFA and risk of stroke with available data from prospective cohort studies. The novelty of the present study was to quantitatively evaluate associations between individual LC n-3 PUFA as biomarker and risk of stroke and fill in the gap that lacks detailed association for subtypes of stroke.

## RESULTS

### Literature search

**Figure 1:**
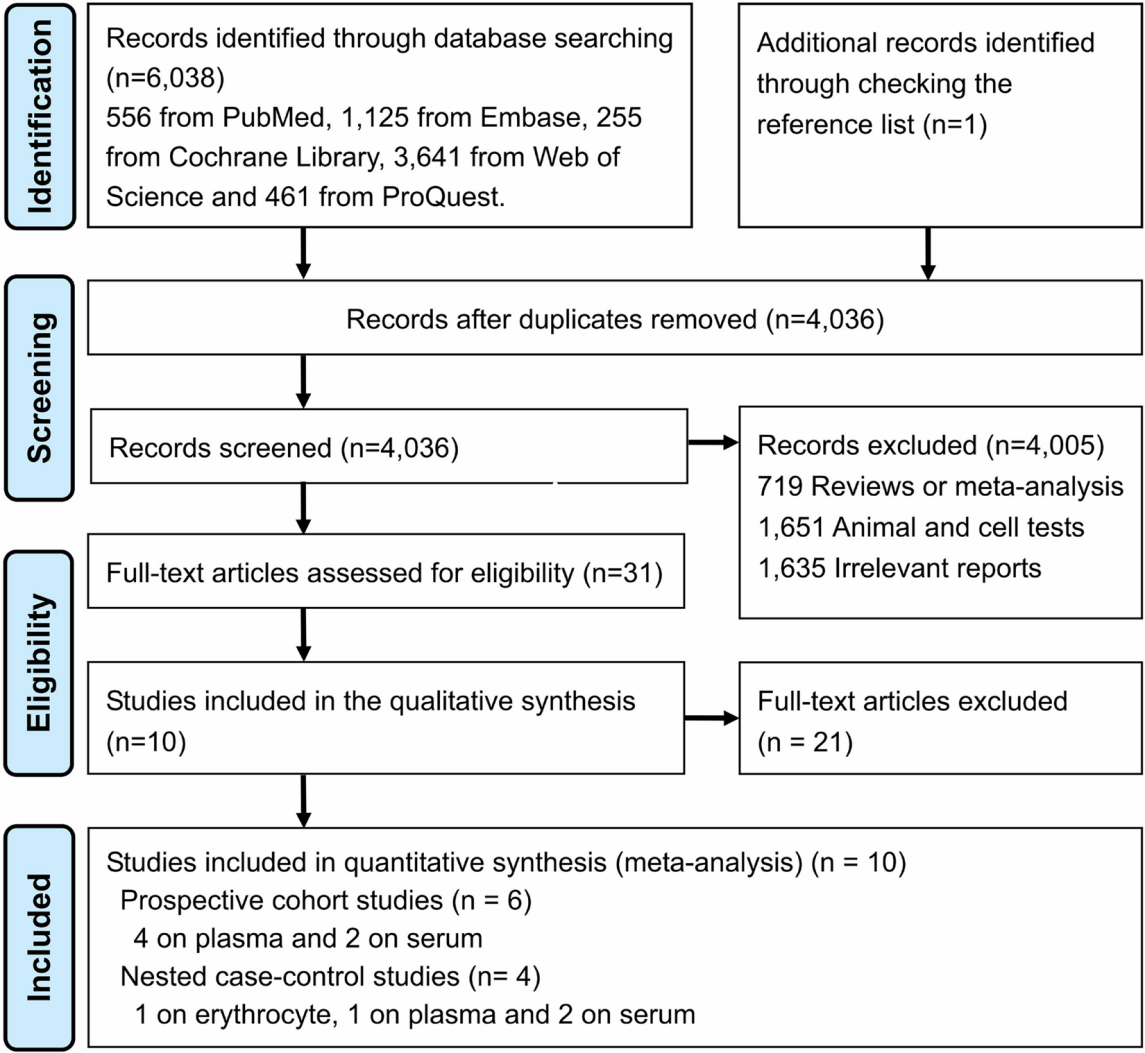
PRISMA flow diagram for included prospective cohort studies

In total, 1,396 unique citations were identified from electronic search plus 2 additional articles retrieved from reference lists (Figure 1). After the titles and abstracts were screened, 28 articles were eligible for further full-text review. Ten relevant studies were finally included in this meta-analysis, and 18 published investigations were discarded. because type of study did not correspond to prospective cohorts, n-6 PUFA were measured only, authors did not provide data of circulating LC n-3 PUFA or data did not correspond to IS or HS.

### Baseline characteristics

Overall, 1 study provided hazard ratio (HR) for plasma LC n-3 PUFA in relation to mortality of stroke only [8], and thus 10 prospective studies (6 cohort and 4 nested case-control studies) that assessed circulation LC n-3 PUFA as a biomarker in relation to incidence of stroke were eligible for the final analysis. Over the 11 years of median follow-up duration, which ranged from 4.7 to 29.3 years, a total of 2,836 stroke events occurred among 20,460 individuals aged 35–79 yr from Europe and US (Table 1). Among the 10 included studies, 5 studies were conducted in USA [8–12] and 5 in Europe [13–17]. Circulating total LC n-3 PUFA as a biomarker was provided in 9 studies [8–17], 20:5n-3 in 8 studies [8–13, 16, 17], 22:5n-3 in 6 studies [8–10, 12, 16, 17], and 22:6n-3 in 8 studies [8–13, 16, 17]. Of those, serum/plasma proportions of LC n-3 PUFA were determined in 9 studies and erythrocytes in 1 study only [14]. The proportions of LC n-3 PUFA in blood samples were quantified by gas liquid chromatography (GLC). One study separately included males (M) and females (F) [14], 3 articles only M [9, 13, 17], 1 study [10] only F and 5 studies included both M and F. Study quality assessed by the 9-star Newcastle-Ottawa Scale (NOS) ranged from 6 to 8, with a median of 7. All included studies provided relative risk (RR) that was controlled for age, gender, smoking and alcohol intake, 8 studies adjusted for lifestyle plus traditional cardiovascular disease (CVD) risk factors [8, 10–15, 17], and 3 studies additionally adjusted for dietary intakes [8, 10, 12].

**Table 1:** Baseline characteristics of individual prospective cohort studies

Reference	Study name (location)	Study design (cases/subjects)	Age (media, yr) and gender	Follow-up duration (median, years)	Exposure of interest		Outcomes		Covariates adjusted ^a^	Quality scores
					Measurement (biomarkers)	Exposure range	Endpoint	RR (95% CI)		
Simon et al., 1995	CHDPPT (US)	NCC (96/192)	46, Male	6.90	GLC (Serum PL)	Mean (SD) in controls		Per a SD increment:	+	7
					GLC (Serum PL)	20:5n-3: 0.71 (0.42)	TS	1.00 (0.73–1.36)		
					GLC (Serum PL)	22:5n-3: 1.03 (0.25)	TS	0.78 (0.56–1.09)		
					GLC (Serum PL)	22:6n-3: 3.24 (1.17)	TS	0.94 (0.70–1.27)		
Wiberg et al., 2006	ULSMA (Sweden)	Cohort (421/2,313)	50, Male	29.30	GLC (Serum CE)	Mean (SD) in controls		Per a SD increment:	++	8
					GLC (Serum CE)	20:5n-3: 1.3 (0.6)	TS	1.04 (0.93–1.15)		
					GLC (Serum CE)	22:6n-3: 0.7 (0.2)	TS	1.01 (0.91–1.12)		
					GLC (Serum CE)	20:5n-3: 1.3 (0.6)	TS	1.05 (0.93–1.19)		
					GLC (Serum CE)	22:6n-3: 0.7 (0.2)	TS	1.01 (0.89–1.14)		
					GLC (Serum CE)	20:5n-3: 1.3 (0.6)	HS	1.07 (0.85–1.34)		
					GLC (Serum CE)	22:6n-3: 0.7 (0.2)	HS	1.04 (0.83–1.30)		
Wennberg et al., 2007	MONICA (Sweden)	NCC (96/192)	55	15.75	GLC (Erythrocyte)	Mean (SD) in controls		Q_4_ vs. Q_1_	++	
			Male		GLC (Erythrocyte)	LC n-3: 5.61 (1.33)	TS	1.08 (0.92–1.28)		
			Female		GLC (Erythrocyte)	LC n-3: 5.88 (1.43)	IS	0.98 (0.81–1.17)		
			Male		GLC (Erythrocyte)	LC n-3: 5.61 (1.33)	IS	1.20 (0.99–1.46)		
Goede et al., 2013	MORGEN (Netherlands)	NCC (179/358)	43, Both (male, 53.00%)	10.50	GLC (Plasma CE)	Mean (SD) in controls		Per a SD increment:	++	
					GLC (Plasma CE)	LC n-3: 1.23 (0.56)	TS	1.16 (0.94–1.45)		
					GLC (Plasma CE)	LC n-3: 1.25 (0.60)	IS	1.33 (0.96–1.84)		
					GLC (Plasma CE)	LC n-3: 1.29 (0.78)	HS	1.08 (0.75–1.57)		
Mozaffarian et al., 2013	CHS (US)	Cohort (406/2,092)	72, Both (male, 36.30%)	11.50	GLC (Plasma PL)	Median (highest quintile) in participants		Q_5_ vs. Q_1_	+++	8
					GLC (Plasma PL)	20:5n-3: 0.92 (0.73–8.52)	TS	1.05 (0.76–1.45)		
					GLC (Plasma PL)	22:5n-3: 1.04 (0.96–1.63)	TS	0.74 (0.55–1.01)		
					GLC (Plasma PL)	22:6n-3: 4.34 (3.76–8.17)	TS	0.84 (0.59–1.18)		
					GLC (Plasma PL)	20:5n-3: 0.92 (0.73–8.52)	TS	1.09 (0.76–1.57)		
					GLC (Plasma PL)	22:5n-3: 1.04 (0.96–1.63)	IS	0.78 (0.55–1.10)		
					GLC (Plasma PL)	22:6n-3: 4.34 (3.76–8.17)	IS	0.74 (0.50–1.10)		
					GLC (Plasma PL)	20:5n-3: 0.92 (0.73–8.52)	HS	0.70 (0.30–1.67)		
					GLC (Plasma PL)	22:5n-3: 1.04 (0.96–1.63)	HS	0.66 (0.32–1.35)		
					GLC (Plasma PL)	22:6n-3: 4.34 (3.76–8.17)	HS	1.24 (0.52–2.94)		
Yaemsiri et al., 20_13_	WHI-OS (US)	NCC (964/964)	64, Female	10.00	GLC (Serum)	Median (quartile range) in controls		Per a SD increment:	+++	7
					GLC (Serum)	20:5n-3: 0.92 (0.73–8.52)	IS	0.89 (0.74, 1.08)		
					GLC (Serum)	22:5n-3: 1.04 (0.96–1.63)	IS	0.75 (0.61, 0.91)		
					GLC (Serum)	22:6n-3: 4.34 (3.76–8.17)	IS	0.76 (0.62, 0.93)		
Yamagishi et al., 2013	ARIC (US)	Cohort (168/3,870)	54, Both (male, 48.03%)	20.00	GLC (Plasma PL)	Median (quartile range) in controls		Q_4_ vs. Q_1_	+	7
					GLC (Plasma PL)	20:5n-3: ND	IS	1.18 (0.78–1.78)		
					GLC (Plasma PL)	22:6n-3: 6.07 (3.26–8.88)	IS	0.69 (0.46–1.06)		
					GLC (Plasma PL)	LC n-3: 9.13 (4.75–13.5)	IS	0.85 (0.55–1.29)		
Virtanen et al., 2013	CHS (US)	Cohort (170/1,056)	72, Both (male, 40.10%)	5.00	GLC (Plasma PL)	Highest quartile in controls		Q_4_ vs. Q_1_	+++	8
					GLC (Plasma PL)	20:5n-3: > 0.70	IS	0.80 (0.47–1.34)		
					GLC (Plasma PL)	22:5n-3: > 0.94	IS	0.84 (0.51–1.39)		
					GLC (Plasma PL)	22:6n-3: > 3.64	IS	0.81 (0.47–1.39)		
					GLC (Plasma PL)	LC n-3: > 5.16	IS	0.77 (0.46–1.31)		
Fezeu et al., 2014	SU.FOL.OM3 (France)	Cohort (85/2263)	62, Both (male, 80.10%)	4.70	GLC (Plasma)	Mean (SD) in no events		Q_4_ vs. Q_1_	++	6
					GLC (Plasma)	20:5n-3: 1.42 (0.87)	IS	0.84 (0.37–1.91)		
					GLC (Plasma)	22:5n-3: 0.60 (0.15)	IS	0.59 (0.26–1.31)		
					GLC (Plasma)	22:6n-3: 2.75 (0.92)	IS	0.65 (0.29–1.47)		
					GLC (Plasma)	LC n-3: 4.77 (1.76)	IS	0.69 (0.30–1.60)		
Daneshmand, et al., 2016	KIHD (Finland)	Cohort (202/1828)	52, Male	21.20	GCL (Serum)	Highest quartile in participants		Q_4_ vs. Q_1_	++	8
					GCL (Serum)	20:5n-3: > 1.97	TS	1.17 (0.76–1.79)		
					GCL (Serum)	22:5n-3: > 0.61	TS	0.99 (0.66–1.48)		
					GCL (Serum)	22:6n-3: > 2.83	TS	1.01 (0.68–1.51)		
					GCL (Serum)	LC n-3: > 5.34	TS	0.90 (0.60–1.34)		
					GCL (Serum)	20:5n-3: > 1.97	IS	1.20 (0.74–1.96)		
					GCL (Serum)	22:5n-3: > 0.61	IS	1.22 (0.77–1.94)		
					GCL (Serum)	22:6n-3: > 2.83	IS	0.99 (0.63–1.57)		
					GCL (Serum)	LC n-3: > 5.34	IS	0.91 (0.58–1.44)		
					GCL (Serum)	20:5n-3: > 1.97	HS	0.83 (0.36–1.91)		
					GCL (Serum)	22:5n-3: > 0.61	HS	0.54 (0.23–1.25)		
					GCL (Serum)	22:6n-3: > 2.83	HS	0.91 (0.41–2.04)		
					GCL (Serum)	LC n-3: > 5.34	HS	0.76 (0.33–1.78)		

Abbreviations: rr: risk ratio; 95% ci: confidence interval; the highest category; sd: standard deviation; ts: total stroke; is: ischemic stroke; hs: hemorrhagic stroke; glc: gas liquid chromatography; pl: phospholipids; ce: cholesterol; the lowest category; 20:5n-3: eicosapentaenoic acid (epa); 22:5n-3: docosapentaenoic acid (dpa);22: 6n-3: docosahexaenoic acid (dha); lc n-3 pufa: long-chain n-3 polyunsaturated fatty acid (20:5n-3 + 22:5n-3 + 22:6n-3); chdppt: a chd primary prevention trial; ulsam: uppsala longitudinal study of adult men; monica: multinational monitoring of trend and determinants in cardiovascular disease; morgen: monitoring project on risk factors for chronic diseases; chs: cardiovascular health study; whi-os: women's health initiative observational study; aric: atherosclerosis risk in community cohort; su.Fol.Om3: supplementation with folate, vitamins b6 and b12 and/or n-3 fatty acids randomized controlled trial; kihd: kuopio ischemic heart disease risk factor study.

^A^ degree of multiple adjustments indicated by +: lifestyle factors (e.G., Age, gender, smoking and alcohol intake); ++: lifestyle plus traditional cvd risk factors (e.G., Bmi, physical activity, blood pressure and blood lipids); +++: lifestyle, traditional cvd risk factors and dietary variables (total energy, fiber and fish intake).

### The top tertiles compared with bottom analyses

**Figure 2:**
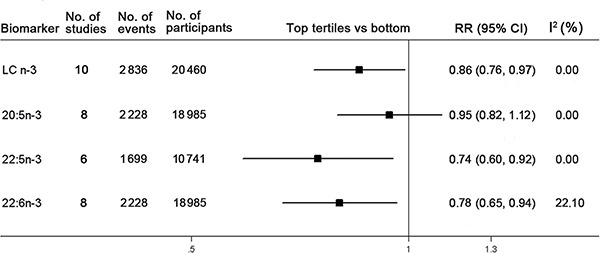
Associations between circulating LC n-3 PUFA and risk of stroke in the highest tertiles compared with the bottom Pooled association estimate concerning circulating total or individual long-chain (LC) n-3 PUFA are referred to by number of included studies, stroke events and participants. The pooled relative risk (RR) estimated by a random-effect model in the highest compared with the bottom tertiles of total or individual LC n-3 PUFA is represented by the black squares, and corresponding confidence interval (CI) is represented by the error bars. The degree of heterogeneity between individual study was indicated by I square statistic.

Overall, 10 prospective cohort studies were eligible for the meta-analysis, comprising 2,836 stroke events and 20,460 participants. The pooled RR estimated by a random-effect model in the highest compared with the bottom tertiles of total or individual LC n-3 PUFA were presented in Figure 2. Participants in the top tertiles of circulating LC n-3 PUFA have a significantly lower risk of stroke compared with those in the bottom (summary RR (SRR) = 0.86; 95% CI: 0.76, 0.97), with no between-study heterogeneity (I^2^ = 0.00%) (Supplementary Figure 1). For individual LC n-3 PUFA, the SRR was 0.95 (95% CI: 0.82, 1.12; *I****^2^*** = 0.00%) for 20:5n-3, 0.74 (95% CI: 0.60, 0.92; *I****^2^*** = 0.00%) for 22:5n-3, and 0.78 (95% CI: 0.65, 0.94; *I****^2^*** = 21.90%) for 22:6n-3, respectively (Supplementary Figures 2, 3 and 4).

**Figure 3:**
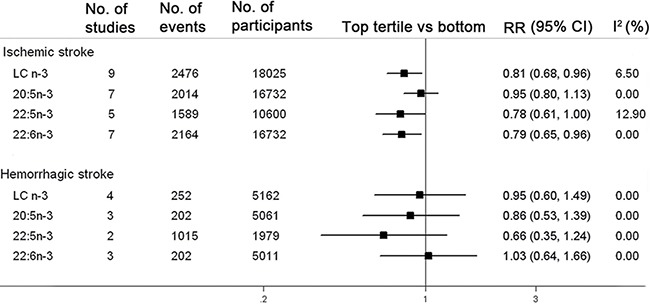
Associations between circulating LC n-3 PUFA and risk of stroke subtypes in the highest tertiles compared with the bottom The pooled association between circulating total or individual LC n-3 PUFA and risk of stroke are subgrouped by the subtypes of stroke. The pooled relative risk (RR) estimated by a random-effect model in the highest compared with the bottom tertiles of total or individual LC n-3 PUFA is represented by the black squares, and corresponding confidence interval (CI) is represented by the error bars. The degree of heterogeneity between individual studies was indicated by I square statistic.

Stratified analyses with meta-regression were performed to detect if the summary RR significantly differed between each strata analyzed (Supplementary Table 3). There was no apparent evidence that the estimated SRR differed significantly by study design, geographic regions, gender, baseline age, follow-up duration, quality scores and biomarker subtypes. Circulating LC n-3 PUFA was inversely associated with incidence of stroke after adjustment for lifestyle, CVD risk factors plus dietary factors (4 studies), whereas no significant association was observed after adjustment for lifestyle and CVD risk factors (5 studies). However, the difference between the two multivariate-adjusted models was not statistically significant with meta-regression. In addition, the associations were more pronounced with ischemic stroke (RR: 0.81; 95% CI: 0.68, 0.96; *n* = 9 studies), but not with hemorrhagic stroke (RR: 0.95; 95% CI: 0.60, 1.49; *n* = 4 studies) (Figure 3). No significant difference was exhibited between the two subtypes of stroke with meta-regressions.

A sensitivity analysis in which summary RR is re-estimated after omitting one study suggested that one study had a substantial influence (Supplementary Figure 5) [10]. After the study was excluded, data from the remaining studies (9 studies) showed the overall result was not significant (SRR = 0.91; 95% CI: 0.79, 1.03). No evidence of publication bias was suggested by visual inspection of Begg's funnel plot (*P* for bias = 0.84) and Egger's regression test (*P* for bias = 0.35) (Supplementary Figures 6 and 7).

### Dose-response analyses

**Figure 4:**
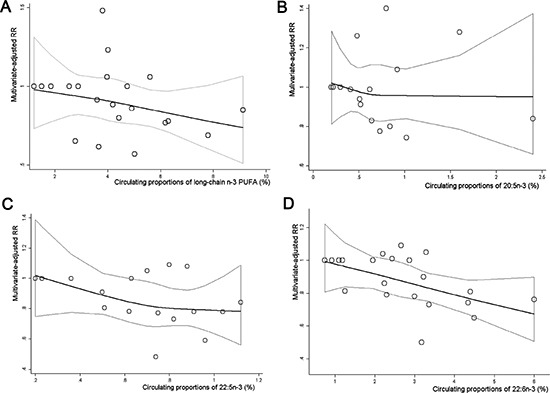
Dose-response association between circulating proportions of LC n-3 PUFA and risk of stroke Multivariate-adjusted relative risks (RRs) from all category of individual or total LC n-3 PUFA in each original study were represented by the small gray circle. The corresponding nonlinear dose-response relationship of total LC n-3 PUFA (**A**), 20:5n-3 (**B**), 22:5n-3 (**C**) and 22:6n-3 (**D**) with risk of total stroke was assessed by a restricted cubic spline model with three fixed knots, and represented by the black solid line, respectively.

Overall, 6 eligible studies were available to evaluate a dose-response association between circulating LC n-3 PUFA and risk of stroke [8, 10–12, 16, 17]. No significantly curvilinear (nonlinear) association was observed through a test for non-linearity (*P* for nonlinearity = 0.40) (Figure 4A), but the association was significant in a linear dose-response model (*P* for trend = 0.02), with a 1% increment of LC n-3 PUFA proportions in circulating blood associated with 5% reduced risk of stroke (SRR = 0.95; 95% CI: 0.92, 0.98; *I^2^* = 14.90%) (Supplementary Figure 8). For the dose-response analysis for individual LC n-3 PUFA (Figure 4B–4D), 5 studies were eligible to conduct the trend estimates for 20:5n-3 and 22:5n-3 [8, 10, 12, 16, 17], while 6 studies were available for 22:6n-3 [8, 10–12, 16, 17]. A significantly linear relationship was found for 22:5n-3 (*P* for nonlinearity = 0.32; *P* for trend = 0.01) and 22:6n-3 (*P* for nonlinearity = 0.07; *P* for trend = 0.01), respectively. Each 1% increment of 22:5n-3 and 22:6n-3 proportions in circulating blood was associated with 25% (SRR = 0.75; 95% CI: 0.64, 0.87; *I^2^* = 1.30%) (Supplementary Figure 9) and 11% (SRR = 0.89; 95% CI: 0.83, 0.95; *I^2^* = 0.00%) reduced risk of stroke (Supplementary Figure 10), respectively. No significantly linear or non-linear dose-response association was found between circulating 20:5n-3 and risk of stroke (Supplementary Figure 11, Figure 4B).

## DISCUSSION

The current study suggests that circulating proportions of LC n-3 PUFA was significantly associated with reduced risk of stroke, especially 22:5n-3 and 22:6n-3. The association was more pronounced with IS, but not with HS. The evidence further builds and extends on prior meta-analyses of LC n-3 PUFA in relation to stroke, and fills in the gap that lacks detailed associations for subtypes of stroke.

Most cohort studies of general populations have assessed self-reported dietary intake of n-3 PUFA rather than objective biomarkers, which may have led to measurement errors or bias. One meta-analysis of 14 prospective cohort studies revealed that LC n-3 PUFA intake was significantly associated with reduced risk of total stroke [2], whereas another one found no significant association for total stroke but did detect a remarkably negative association for IS [3]. It is difficult to objectively identify a remarkable association, due to a relatively low consumption of typical fish or seafood rich in LC n-3 PUFA among the US and Europe populations. Moreover, a meta-analysis of 12 randomized controlled trials suggested that LC n-3 PUFA supplementation (average 1.8 g/day, treatment duration of 3 years) did not reduce IS or HS events [18]. The favorable effects may be diminished if background dietary intake of n-3 PUFA is higher among a sample of study populations. This meta-analysis also included 4 observational studies with circulating LC n-3 PUFA as interest exposure, and found circulating LC n-3 PUFA cannot be associated with total stroke. One potential issue was that this meta-analysis included one individual study with unadjusted results [19] and another study with the incidence of coronary heart disease as end point rather than stroke [20]. Thus, the direction of true association may be changed. Another issue was difficult to evaluate the association for stroke subtypes, mainly due to the limited number of eligible studies. In contrast, the present study quantitatively evaluated the association of circulating total or individual LC n-3 PUFA with risk of stroke and its subtypes, which was performed under a more rigorous inclusion criterion, and provided a sufficient statistical power to achieve a significant result for IS.

The different effect of circulating LC n-3PUFA in relation to the stroke subtype was observed in our study. Circulating LC n-3 PUFA was found to be favorably associated with IS, which was similar to a previous meta-analysis with dietary intake of LC n-3 PUFA as interest exposure [3] and 3 case-control studies with blood levels of LC n-3 PUFA as a biomarker [21–23]. LC n-3 PUFA has presented anti-thrombotic, anti-oxidative and anti-inammatory properties, which could be speculated to alleviate the progression of IS [24]. Recent evidence from animal models have also demonstrated that LC n-3 PUFAs can robustly attenuate ischemic neuronal injury by activating Akt-dependent pro-survival pathway [25] and up-regulating nuclear factor E2-related factor 2 (Nrf2) [26] to promote the biosynthesis of phosphatidylserine in neuronal cell membranes. By contrast, a null association for HS was observed in the present study, and no sufficient evidence was found to support that LC n-3 PUFAs have apparent benefits for HS. Intake of large amounts of n-3 PUFA can reduce blood viscosity and platelet aggregation as well as decreased clotting factors of the intrinsic pathway, which leads to increased bleeding times [27]. These changes may be response for the increased risk of HS in populations with high dietary intake of LC n-3 PUFA [28, 29]. Data from animal models have also shown that increased intake of LC n-3 PUFA can induce oxidative damage to the brain [30] and worsen forelimbs motors function in rats with HS [31].

However, the limited numbers of prospective cohort studies included in our meta-analysis might diminish the statistical power to detect the association for HS, and thus the remarkable evidence may be difficult to be found. Large-scale and well-designed prospective population-based studies are required to further determine whether increased circulating levels of LC n-3 PUFA will be adversely associated with incident HS.

We found that individual circulating LC n-3 PUFA was differentially associated with incidence of stroke, and 22:6n-3 has the greatest effects. Multiple mechanisms have been proposed to mediate neuroprotection by n-3 PUFAs, including the activation of pro-survival signaling cascades in neurons, blunting of microglia-mediated inflammatory responses [32], and by inhibiting interleukin 1β-induced NF-κB activation [33]. Additionally, 22:6n-3 may be preferentially effective in altering lipid membrane structure and attenuating brain necrosis after hypoxic ischemic injury to reach a better stabilizing intracellular ion balance in hypoxic-ischemic insult [34]. Finally, only 22:6n-3 is metabolized to neuroprotectin-D1 (NPD1), which can confer a unique inflammation-resolving property [35]. Furthermore, we also found that circulating 22:5n-3 was strongly associated with reduced risk of IS. A possible explanation is that 22:5n-3 in human platelets is further metabolized into 11- and l4-hydroxy docosapentaenoic and reduces the thromboxane B2 (TXB_2_) production from arachidonic acid through decelerating the cyclooxygenase pathway and accelerating the lipoxygenase pathway to inhibit platelet aggregation and thrombogenesis [36].

The present study has several strengths. Firstly, the prospective study design minimized the possibility of recall, and allowed inference on temporality of associations. In addition, circulating biomarkers can provide objective measures of individual n-3 PUFA. Finally, the association between circulating LC n-3 PUFA and risk of stroke was stratified by pathological stroke subtypes. Nevertheless, our study also has some limitations. Firstly, our search was limited to English publications, and thus non-English or unpublished reports may exist. Secondly, dietary intake and metabolic fluctuations over time could increase exposure misclassification during follow-up. Thirdly, LC n-3 PUFA levels in the brain cannot be precisely represented by circulating FA status, which may attenuate measures of a true association. Fourthly, relatively few HS events occurred, limiting statistical power for this end point. Fifthly, postmenopausal women in a nested case-control study might not be an unbiased representative of a general population [10], thus selection bias cannot be minimized. However, sensitivity analyses indicated that the overall result was still marginally significant when the individual study [10] was omitted from the analysis. Sixthly, each original study reported RR calculated by different multivariable models, thus the direction of summary results may partially be changed. However, subgroup analysis showed that the pooled association remained significant after adjustment for lifestyle, traditional CVD risk factors and dietary intake. Finally, despite comprehensive adjustment for confounders in each original study, we cannot exclude the possibility of residual confounding caused by imprecisely measured or unmeasured factors.

In summary, the circulating proportion of LC n-3 PUFA is linearly associated with reduced risk of stroke, especially 22:6n-3 and 22:5n-3. Low levels of circulating LC n-3 PUFA may be an independent risk factor for stroke, especially for IS. If these observations can be further confirmed in large-scale prospective intervention studies, encouraging the consumption of food rich in LC n-3 PUFAs to ultimately improve their circulating levels may offer a potential public health benefit for ischemic stroke.

## MATERIALS AND METHODS

### Literature research

We followed MOOSE guidelines of observational studies for conducting and reporting the present meta-analysis (Supplementary Table 1) [37]. Systematic literature searches were conducted to identify prospective cohort studies that reported the association between circulating LC n-3 PUFA and risk of stroke from EMBASE (http://www.embase.com, since 1947), the Cochrane Library (http://www.thecochranelibrary.com/, since 1951), Web of science (http://isiknowledge.com, since 1961), ProQuest (http://search.proquest.com/, since 1961) and PubMed.

(http://www.ncbi.nlm.nih.gov/pubmed, since 1966) up to June 2017, respectively. The literature strategy was performed using a method of the key words combined with medical subject headings, and the full details are presented in Supplementary Method. Our search was restricted to human studies, without the limitation of the language and publication status, while the meeting abstracts and duplicated studies were excluded. We did not contact authors for the detailed information of primary studies and unpublished studies. We also searched systematic reviews from the above-mentioned databases, and checked the reference lists to identify original studies that might have been missed.

### Eligibility criteria

The relevant studies were included in the meta-analysis if they met the following inclusion criteria: (1) Participants: Adults of any age across different countries; (2) Exposure of interest: Quantitative determination of total or individual LC n-3 PUFA (20:5n-3, 22:5n-3 and 22:6n-3) in circulating blood (serum/plasma/whole blood/erythrocytes); (3) Outcomes: Evaluating incidence and/or mortality of stroke or its specific subtypes (IS and HS) as an end point and reporting multivariate-adjusted relative risk (RR) or hazard ratio (HR) with 95% confidence intervals (CI); and (4) Study design: Prospective cohort study (cohort, nested case-control and case-cohort study).

### Data extraction

Data extraction was completed independently and performed twice by two investigators, and disagreements were reconciled by consensus. The following data was extracted from each original study: study name (location), size (number of cases/participants) and types of prospective studies (cohort vs. nested case-control), baseline age (median, yr) and gender, duration of follow-up (median, years), exposure of interest (assay methods, exposure range), subtypes of stroke (IS vs. HS) and multivariate-adjusted RR (HR) with 95% CI for all categories of circulating LC n-3 PUFA and degree of adjustment for the potential confounders. Adjustment for lifestyle factors (baseline age, gender, race, smoking and alcohol intake) was defined as “+”, “++” for lifestyle factors plus other cardiovascular disease (CVD) risk factors (e.g., BMI, physical activity, blood pressure and blood lipids) and “+++” for lifestyle, other CVD risk factors plus dietary factors (e.g., vegetables, dietary fiber and fish intake). Study quality was evaluated by using the 9-stars Newcastle-Ottawa Scale [38]. High or low quality of each study was defined based on the median of overall quality scores among all included studies (Supplementary Table 2).

### Data synthesis

Circulating LC n-3 PUFA was defined as the sum of 22:6n-3, 22:5n-3 and 20:5n-3 proportions in circulating blood (serum/plasma/whole blood/erythrocytes). If an original study reported RR for IS only, the corresponding RR was to approximately represent a RR for total stroke, due to the few HS events which occurred. If an original study provided HR with 95% CI for incidence of stroke, the HR was assumed to approximate RR. All included studies provided RR for biomarker of LC n-3 PUFA based on various categories (e.g., tertiles, quartiles or quintiles,) and/or per SD difference in exposure. Thus, to provide a consistent approach to the meta-analysis, the RRs (HRs) were transformed to involve comparisons between the top and the bottom tertiles of baseline circulating proportions of LC n-3 PUFA by using methods previously described [39, 40]. Briefly, log risk estimates were transformed with the comparison between top and bottom tertiles being equivalent to 2.18 times the log risk ratio for a 1-SD increase [39, 40]. These scaling methods assume that the exposure is normally distributed and that the association with risk of stroke is log-linear. The conversion factor of 2.18 is the difference in the medians of the top and bottom thirds of the standard normal distribution; other conversions were used for differences in medians of extreme quartiles (2.54) or quintiles (2.80) [39, 40]. The standard errors (SEs) of log RRs were calculated using reported data on precision and were similarly standardized [39, 40].

### Statistic analysis

Statistical analyses of the combined data were performed by STATA version 11.0 (Stata CORP, College Station, TX). Two types of meta-analyses were conducted. Firstly, we performed a meta-analysis for the top compared with the bottom tertiles of LC n-3 PUFA. Each multivariate-adjusted RR (HR) for the top compared with the bottom tertiles to assess the association between circulating LC n-3 PUFA and risk of stroke was firstly transformed to their logarithm (logRR), and the corresponding 95% CI was used to calculate the standard error (selogRR). Summary RR (SRR) with corresponding 95% CI as the overall risk estimate for all included prospective cohort studies was calculated using a random-effects model described by DerSimonian and Laird [41], which considers both within-study and between-study variability. Heterogeneity across studies was evaluated with the *Q* test and *I^2^* statistic [42]. We defined an I^2^ value greater than 50% as indicative of heterogeneity according to Cochrane Handbook. Stratified analysis was performed to identify the possible sources of heterogeneity by study design (nested case-control vs. cohort), different regions (Europe vs. US), gender, baseline age (≤ 55 vs. > 55), follow-up duration (≤ median vs. > median), quality scores (≤ 7 vs. > 7), biospecimen types (erythrocyte vs. serum vs. plasma), stroke subtypes (IS vs. HS) and multiple adjustments (lifestyle and CVD risk factors vs. lifestyle and CVD risk factors plus dietary factors). The median of follow-up duration in the present meta-analysis could approximately be represented by calculating the mean of the middle two medians of follow-up duration from the included studies. A univariate meta-regression with restricted maximum likelihood was performed to measure if summary RR significantly differed between each strata analyzed. Sensitivity analysis was performed to evaluate the possible influence of individual study on the summary results. The possibility of publication bias was quantitatively evaluated by Begg's test and Egger's regression test [43].

Additionally, dose-response meta-analyses were conducted to determine a potential curvilinear (nonlinear) or linear association between circulating LC n-3 PUFA and risk of stroke. A curvilinear trend was tested by using methods previously described [44, 45]. Restricted cubic splines with 3 knots (2 spline transformations) at fixed percentiles (25%, 50%, and 75%) was firstly created, and then a *P* for nonlinearity was calculated to detect potential departure from a simpler linear trend by testing the coefficient of the second spline equal to zero [46]. In the presence of substantial linear trends (*P* for nonlinearity > 0.05), a linear trend was estimated to achieve the association between per 1% increment of LC n-3 PUFA proportions in circulating blood and the risk of stroke by using a method of a generalized least-squares regression (2-stage GLST in Stata) [45]. Two-tailed *P* < 0.05 was considered statistically significant.

## SUPPLEMENTARY MATERIALS FIGURES AND TABLES




