# Supplementary material for: Circulating long-chain n-3 polyunsaturated fatty acid and incidence of stroke: a meta-analysis of prospective cohort studies
Source: Oncotarget. 2017 Jul 25;8(48):83781–91. doi: 10.18632/oncotarget.19530 (PMC5663554; doi:10.18632/oncotarget.19530)
Supplement: Supplementary file 1 [file oncotarget-08-83781-s001.pdf]

Circulating long-chain n-3 polyunsaturated fatty acid and incidence of stroke: a meta-analysis of prospective cohort studies

SUPPLEMENTARY MATERIALS

SUPPLEMENTARY METHODS

Literature search strategies.

Literature search strategy in pubmed: 556 publications till 2017 Jun

#1 Search ((((((((Omega 3 Fatty Acids) OR n-3 Polyunsaturated Fatty Acid) OR n 3 Fatty Acids) OR "Fatty Acids, Omega-3"[Mesh]) OR Docosapentaenoic acid[Title/Abstract]) OR Docosahexaenoic acid[Title/Abstract]) OR Eicosapentaenoic acid[Title/Abstract]) OR marine fatty acid[Title/Abstract]) OR dietary fat[Title/Abstract]

#2 Search ((((((Apoplexy) OR brain vascular accident) OR Cerebrovascular accident) OR "Stroke"[Mesh]) OR stroke[Title/Abstract]) OR transient ischemic attack[Title/Abstract]) OR TIA[Title/Abstract]

#1 and #2

Literature search strategy in embase: 1,125 publications till 2017 Jun

#1 'omega 3 fatty acid'/exp
#2 'eicosapentaenoic acid':ab
#3 'docosapentaenoic acid':ab
#4 'docosahexaenoic acid':ab
#5 'marine fatty acid':ab
#6 'dietary fat':ab
7 #1 OR #2 OR #3 OR #4 OR #5 OR #6
8 'cerebrovascular accident'/exp
#9 'stroke':ab
#10 'transient ischemic attack':ab
#11 'TIA':ab
#12 'apoplexy':ab
13 #8 OR #9 OR #10 OR #11 OR #12
#14 #7 and #13

Literature search strategy in cochrane library: 255 publications till 2017 Jun

#1 MeSH descriptor: [Fatty Acids, Omega-3] explode all trees

#2 "marine fatty acid":ti,ab,kw or "dietary fat":ti,ab,kw or "eicosapentaenoic acid":ti,ab,kw or "docosahexaenoic acid":ti,ab,kw or "docosapentaenoic acid":ti,ab,kw or "n 3":ti,ab,kw or "omega 3":ti,ab,kw or "PUFA":ti,ab,kw (Word variations have been searched)

#3 MeSH descriptor: [Stroke] explode all trees

#4 Cerebrovascular:ti,ab,kw or "brain vascular accident":ti,ab,kw or "transient ischemic attack":ti,ab,kw or "TIA":ti,ab,kw or "stroke":ti,ab,kw or "apoplexy":ti,ab,kw (Word variations have been searched)

#5 #1 OR #2
#6 #3 OR #4
#7 #5 AND #6

Literature search strategy in web of science: 3,641 publications till 2017 Jun

#1 Subject: ("marine fatty acid") OR ("dietary fat") OR ("omega 3") OR ("n-3") OR ("eicosapentaenoic acid") OR ("docosapentaenoic acid") OR ("docosahexaenoic acid") OR ("PUFA") OR ("fatty acid")

#2 Subject: ("cerebrovascular") OR ("Stroke") OR ("TIA") OR ("transient ischemic attack") OR ("apoplexy").

#3 #1 AND #2

Literature search strategy in proquest: 462 publications till 2017 Jun

(su.Exact("stroke") OR ab("transient ischemic attack") OR ab("apoplexy") OR ab("cerebrovascular") OR ab("TIA")) AND (su.Exact("fatty acids, omega-3") OR ab("dietary fat") OR ab("marine fatty acid") OR ab("n 3") OR ab("omega 3") OR ab(PUFA) OR ab("eicosapentaenoic acid") OR ab("docosapentaenoic acid") OR ab"fatty acid" OR ab("docosahexaenoic acid"))

Supplementary Table 1: Moose checklist of present meta-analysis. See Supplementary_Table_1.

Supplementary Table 2: Quality assessment of included prospective cohort studies by newcastle-ottawa scale (stars)

Study design	Selection(☆☆☆☆)	Comparability(☆☆)	Exposure or Outcome (☆☆☆)	Stars	Quality scores
Cohort studies	1) Representativeness of the exposed cohort? ☆<br>2) Selection of the non exposed cohort? ☆<br>3) Evaluating exposure? ☆<br>4) Outcomes of interest were not present at study start? ☆	1) Study controls for the most important factor? ☆<br>2) Study controls for any additional factors? ☆	1) How to ascertain outcome? ☆<br>a) Independent blindness<br>b) record linkage<br>2) Follow-up till outcomes happened? ☆<br>Adequacy of follow up? ☆	☆☆☆☆☆☆☆☆☆ (9)	High quality: 8–9stars,<br>Moderate quality: 6–7stars,<br>Low quality:1–5 stars
Nested case-control	1)Adequate case definition? ☆<br>2) Representativeness of the cases? ☆<br>3) Community controls? ☆<br>4) Controls have no history of endpoint disease? ☆	1) Study controls for the most important factor? ☆<br>2) Study controls for any additional factors? ☆	1) How to ascertain exposure?☆<br>a) By secure record<br>b)Structured interview where blind to case/control status<br>2) Same method of ascertainment for both? ☆<br>3) Same response rate for both? ☆		
Included cohort studies					
Wiberg et al, 2006 (Sweden)	1) ☆, 2) ☆, 3) ☆, 4) ☆	1) ☆, 2) ☆	1) ☆, 2) ☆, 3) × ☆no description	☆☆☆☆☆☆☆☆	High
Mozaffarian et al, 2013 (US)	1) ☆, 2) ☆, 3) ☆, 4) ×:no statement	1) ☆, 2) ☆	1) ☆, 2) ☆, 3) ☆	☆☆☆☆☆☆☆☆	High
Yamagishi et al, 2013 (US)	1) ☆; 2) ☆, 3) ☆, 4) ☆	1) ☆; 2) ×: no control for additional factors	1) ☆, 2) ☆, 3) × ☆no description	☆☆☆☆☆☆☆	Moderate
Virtanen et al, 2013 (US)	1) ☆, 2) ☆, 3) ☆, 4) ☆	1) ☆; 2) ☆	1) ☆, 2) ☆, 3) ×: no statement	☆☆☆☆☆☆☆☆	High
Fezeu et al, 2014 (France)	1) ×: subjects with coronary or cerebral ischemic event history, 2) ☆, 3) ☆,4) ×:no statement	1) ☆; 2) ☆	1) ☆, 2) ☆, 3) ×: no statement	☆☆☆☆☆☆	Moderate
Daneshmand et al, 2016 (Finland)	1) ×: men cohort, 2) ☆, 3) ☆, 4) ☆	1) ☆; 2) ☆	1) ☆, 2) ☆, 3) ☆	☆☆☆☆☆☆☆☆	High
Included Nested Case-Control Studies					
Simon et al, 1995 (US)	1) ☆, 2) ☆,<br>3) ☆, 4) ×: no statement;	1) ☆<br>2) ×: without covariates adjusted	1) ☆, 2) ☆, 3) ☆	☆☆☆☆☆☆☆	Moderate
Wennberg et al, 2007 (Sweden)	1) ☆, 2) ☆,<br>3) ☆ 4) ×: no mention	1) ☆, 2) ☆	1) ☆, 2) ☆, 3) ☆	☆☆☆☆☆☆☆☆	High
Goede et al, 2013 (Netherlands)	1) × :no mention, 2) ☆,<br>3) ☆ 4) ×: no mention	1) ☆, 2) ☆	1) ☆, 2) ☆, 3) ☆	☆☆☆☆☆☆☆	Moderate
Yaemsiri et al, 2013 (US)	1) ×:record linkage; 2) ☆, 3) ☆, 4) ×: no mention	1) ☆, 2) ☆	1) ☆, 2) ☆, 3) ☆	☆☆☆☆☆☆☆	Moderate

Supplementary Table 3: Subgroup analyses for circulating proportions of LC n-3 PUFA in the top tertiles compared with the bottom

Factors stratified	20:5n-3					22:6n-3					LC n-3 PUFA				
	N	RR (95% CI)	Heterogeneity		P[2]	*N*	RR (95% CI)	Heterogeneity		P[2]	*N*	RR(95% CI)	Heterogeneity		P[2]
			I^2 (%)	P[1]				I^2 (%)	P[1]				I^2 (%)	P[1]	
Overall analysis	8	0.94 (0.79, 1.11)	0.00	0.77		8	0.76 (0.62, 0.92)	0.00	0.56		10	0.86 (0.76, 0.98)	0.90	0.43	
Study design					0.16					0.21					0.78
NCC	2	0.79 (0.59, 1.05)	0.00	0.62		2	0.85 (0.71, 1.06)	4.50	0.30		4	0.90 (0.73, 1.11)	50.80	0.11	
CH	6	1.02 (0.83, 1.25)	0.00	0.89		6	0.64 (0.43, 0.94)	0.00	0.78		6	0.86 (0.74, 1.00)	0.00	0.86	
Regions					0.46					0.29					0.08
Europe	3	1.04 (0.75, 1.44)	0.00	0.58		3	0.95 (0.69, 1.31)	0.00	0.34		5	1.05 (0.83, 1.34)	0.00	0.76	
US	5	0.89 (0.73, 1.07)	0.00	0.67		5	0.75 (0.60, 0.94)	0.00	0.63		5	0.75 (0.62, 0.92)	0.00	0.68	
Gender					0.33					0.31					0.14
Females	1	0.78 (0.57, 1.05)	.	.		1	0.55 (0.34, 0.88)	.	.		2	0.72 (0.51, 1.01)	23.00	0.25	
Males	3	1.08 (0.77, 1.51)	0.00	0.87		3	0.96 (0.71, 1.35)	0.00	0.63		4	1.05 (0.78, 1.41)	0.00	0.74	
Both	4	0.99 (0.71, 1.28)	0.00	0.81		4	0.80 (0.62, 1.03)	0.00	0.90		5	0.84 (0.65, 1.08)	0.00	0.97	
Age (y)					0.17					0.34					0.07
≤ 55	4	1.10 (0.83, 1.10)	0.00	0.96		4	0.90 (0.69, 1.18)	0.00	0.52		6	0.84 (0.65, 1.08)	0.00	0.80	
> 55	4	0.85 (0.69, 1.04)	0.00	0.71		4	0.74 (0.58, 0.95)	0.00	0.48		4	0.74 (0.60, 0.91)	0.00	0.65	
Follow-up (median, years)					0.09					0.23					0.12
≤ 17.0	4	0.81 (0.64, 1.03)	0.00	0.96		4	0.69 (0.52, 0.92)	0.00	0.63		5	0.74 (0.59, 0.93)	0.00	0.44	
> 17.0	4	1.08 (0.86, 1.37)	0.00	0.94		4	0.90 (0.71, 1.13)	0.00	0.52		5	0.98 (0.69, 1.20)	0.00	0.82	
Quality scores					0.35					0.14					0.18
≤7	4	0.87 (0.68, 1.10)	0.00	0.63		4	0.66 (0.50, 0.88)	0.00	0.76		5	0.75 (0.60, 0.95)	1.70	0.39	
> 7	4	1.10 (0.80, 1.29)	0.00	0.70		4	0.93 (0.73,1.18)	0.00	0.75		5	0.97 (0.79, 1.19)	0.00	0.77	
Biospecimen subtypes					0.75					0.98					0.41
Serum	4	0.99 (0.77, 1.28)	0.00	0.82		4	0.80 (0.55, 1.15)	63.00	0.04		4	0.79 (0.63, 0.99)	19.00	0.24	
plasma	4	0.94 (0.77, 1.15)	0.00	0.41		4	0.80 (0.62, 1.02)	0.00	0.90		5	0.89 (0.74, 1.06)	0.00	0.74	
Erythrocyte	ND	ND	ND	ND		ND	ND	ND	ND		1	1.08 (0.70, 1.64)	.	.	
Stroke subtypes					0.89					0.25					0.27
IS	7	0.93 (0.78, 1.12)	0.00	0.64		7	0.77 (0.63, 0.94)	0.00	0.54		9	0.82 (0.68, 0.99)	18.10	0.32	
HS	3	0.97 (0.55, 1.59)	0.00	0.46		3	1.13 (0.65, 1.97)	0.00	0.79		4	1.14 (0.69, 1.91)	0.00	0.89	
Multiple adjustments					0.96					0.35					0.16
Lifestyle and CVD risk factors	4	0.94 (0.76, 1.15)	0.00	0.77		3	0.90 (0.69, 1.18)	0.00	0.60		5	0.98 (0.80, 1.19)	0.00	0.60	
Lifestyle, CVD risk factors and dietary factors	4	0.95 (0.71, 1.27)	0.00	0.43		5	0.74 (0.58, 0.95)	0.00	0.52		5	0.79 (0.67, 0.93)	0.00	0.42	

N, number of included studies; CH, cohort sutdy; NCC, nested case-control study; RR, risk ratio; CI, confidence interval; IS, ischemic stroke; HS, hemorrhagic stroke; ND: No data.
[1]P for heterogeneity within each subgroup with *Q* test.
[2]P for difference between subgroups with meta-regression analysis.

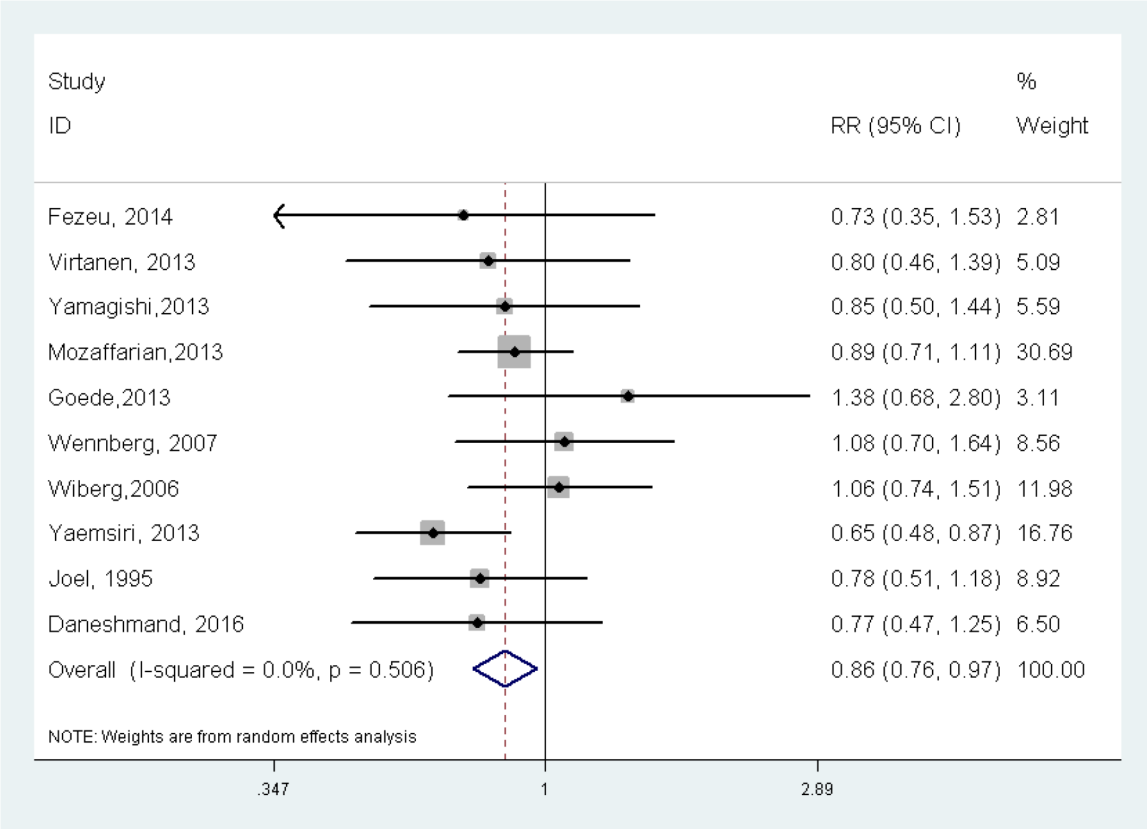

Supplementary Figure 1: Forest plot of association between circulating total LC n-3 PUFA and risk of total stroke in the top tertiles compared with the bottom.

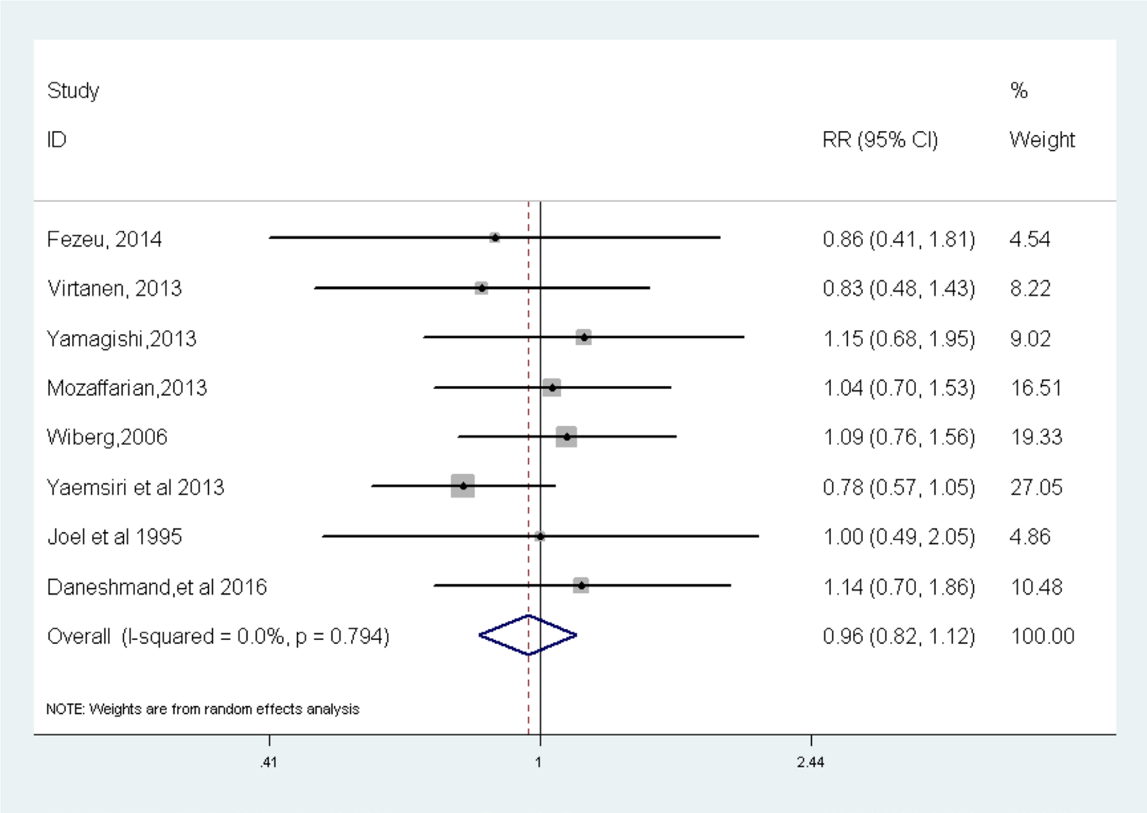

Supplementary Figure 2: Forest plot of association between circulating 20:5n-3 and risk of total stroke in the top tertiles compared with the bottom.

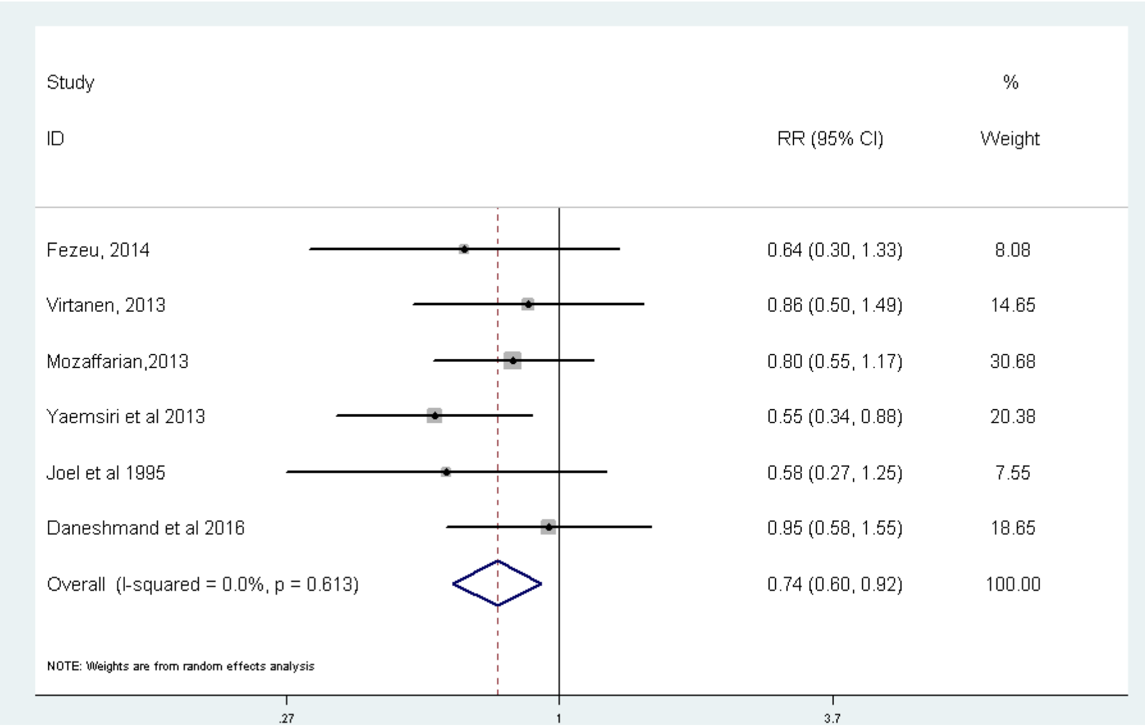

Supplementary Figure 3: Forest plot of association between circulating 22:5n-3 and risk of total stroke in the top tertiles compared with the bottom.

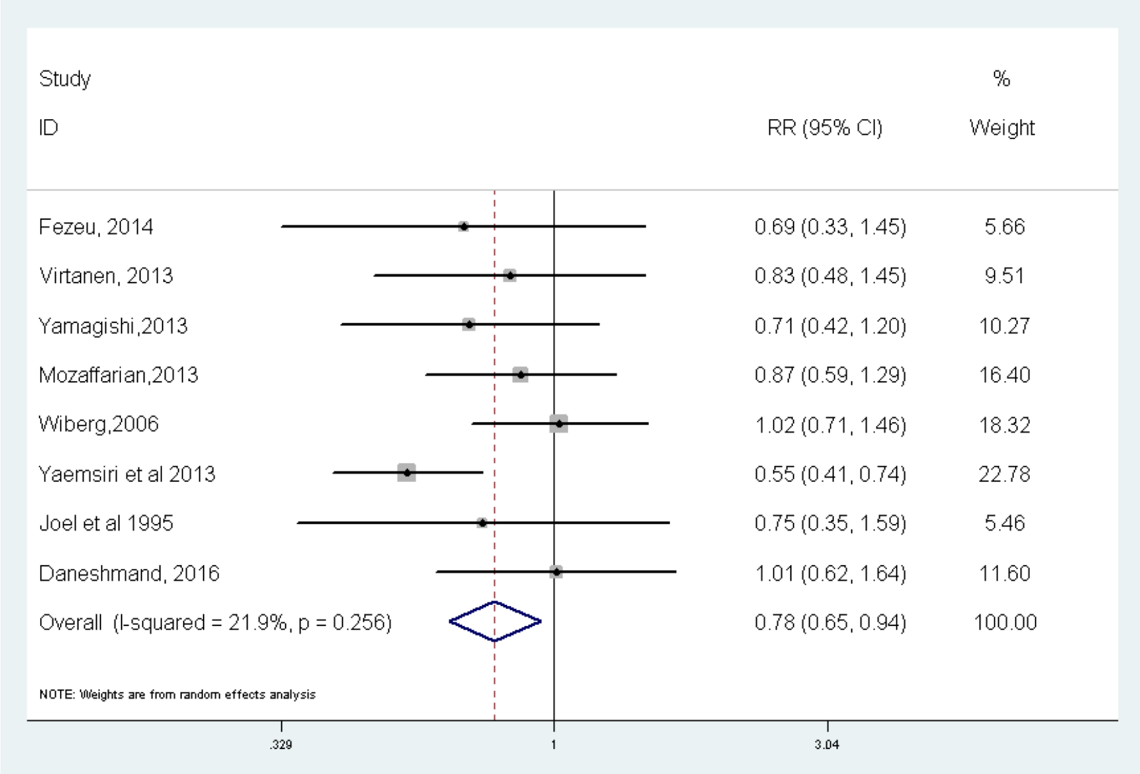

Supplementary Figure 4: Forest plot of association between circulating 22:6n-3and risk of total stroke in the top tertiles compared with the bottom.

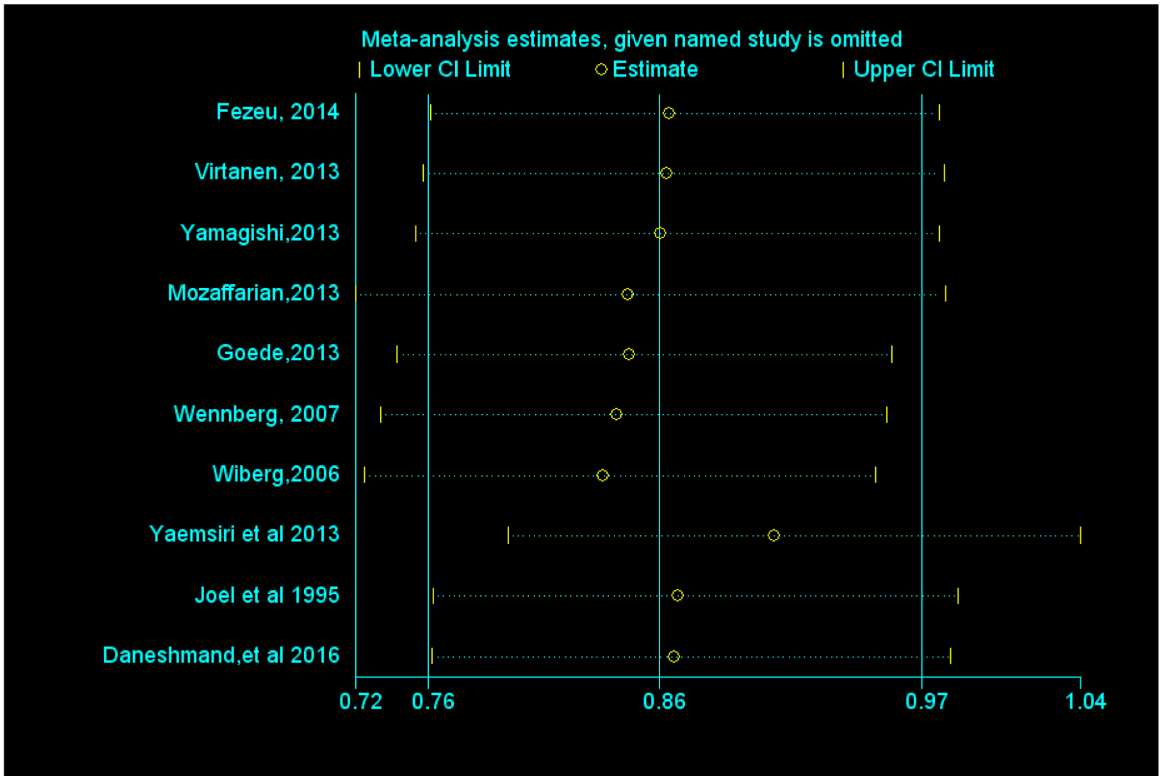

Supplementary Figure 5: Sensitivity analysis on biomarker of LC n-3 PUFA in relation to incidence of stroke in which the pooled relative risk is re-estimated after omitting one study.

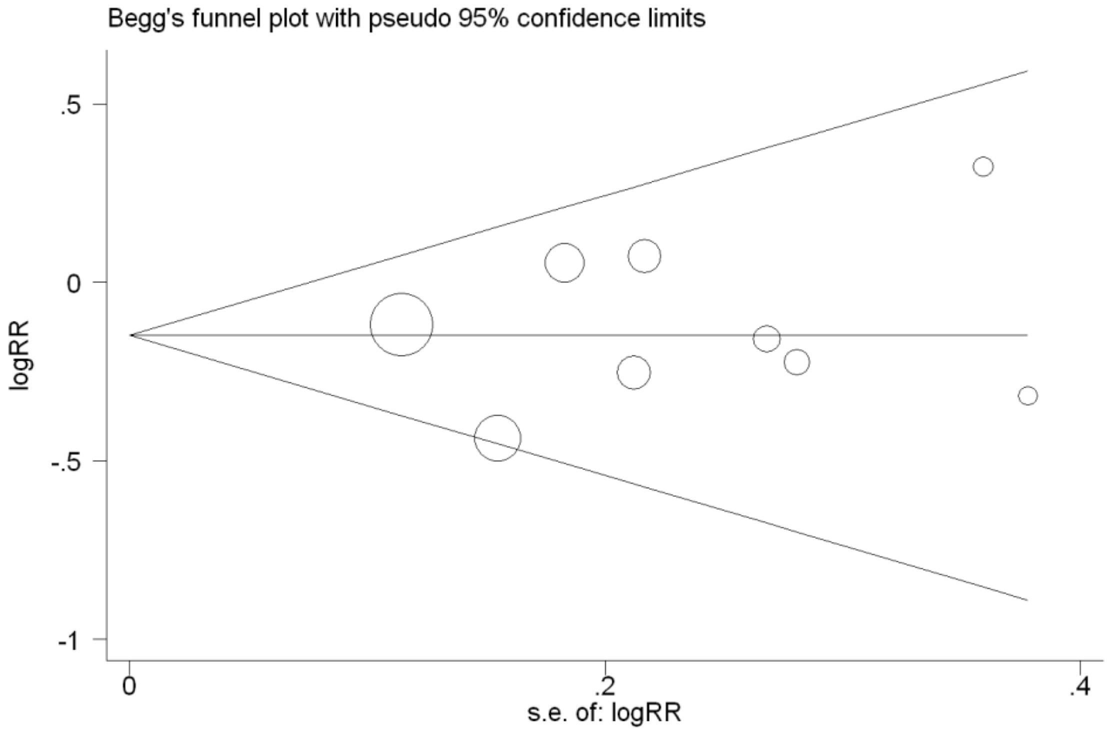

Supplementary Figure 6: Begg's funnel plot on LC n-3 PUFA biomarker with risk of stroke.

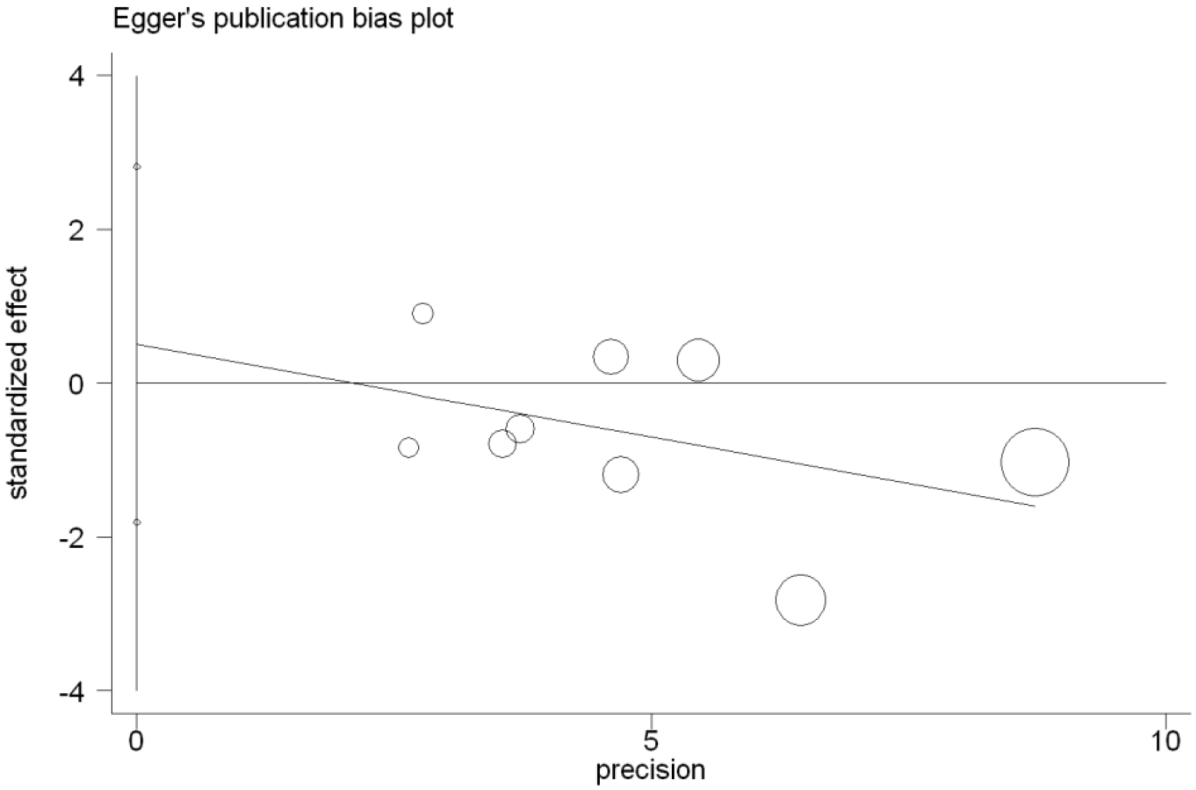

Supplementary Figure 7: Egger's regress plot on LC n-3 PUFA biomarker with risk of stroke.

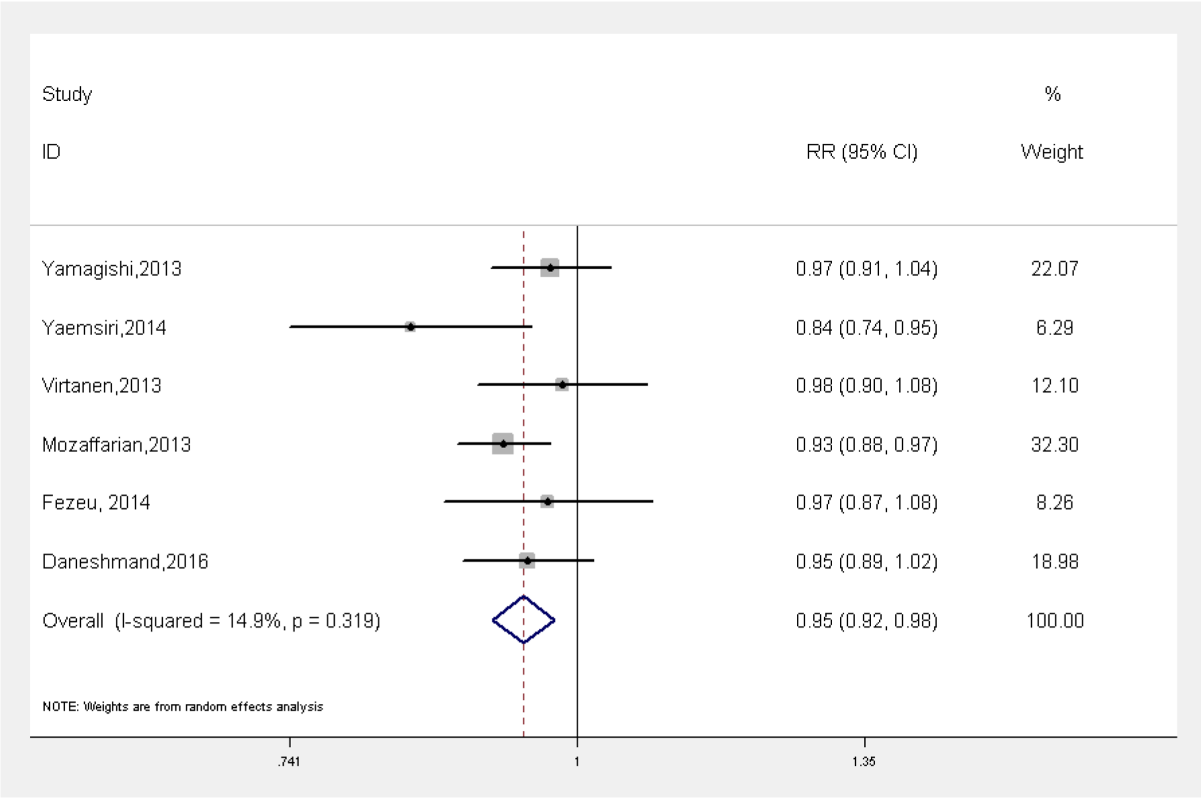

Supplementary Figure 8: Two-stage dose-response association of each 1% increment of LC n-3 proportions in circulating blood with risk of stroke.

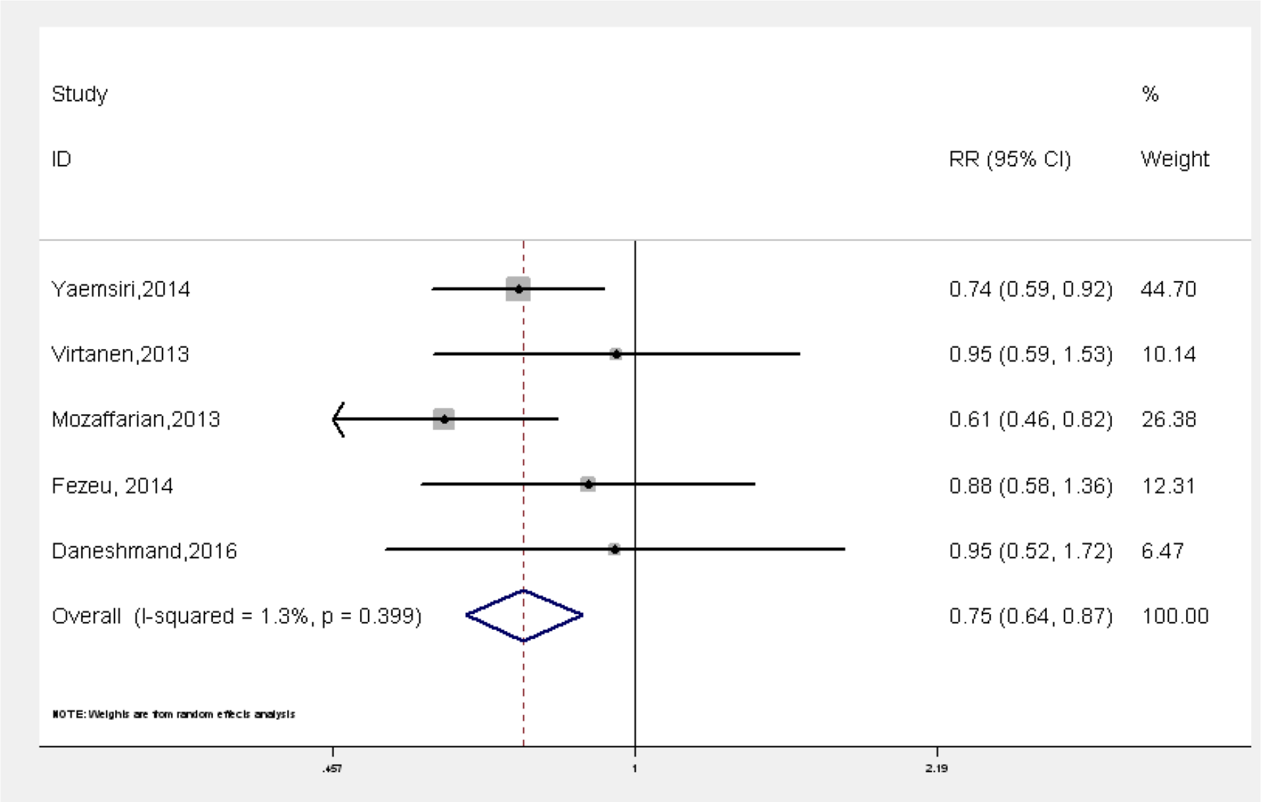

Supplementary Figure 9: Two-stage dose-response association of each 1% increment of 22:5n-3 in circulating blood with risk of stroke.

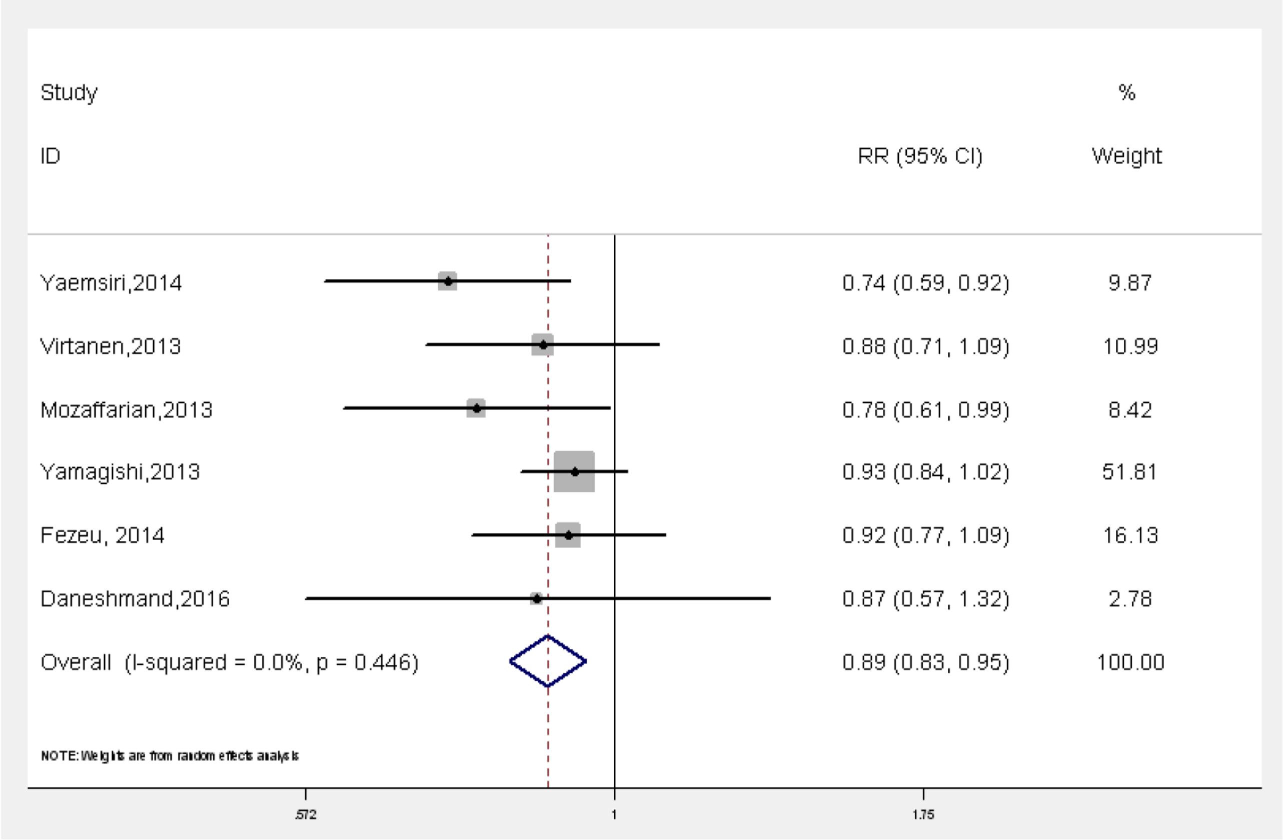

Supplementary Figure 10: Two-stage dose-response association of each 1% increment of 22:6n-3 in circulating blood with risk of stroke.

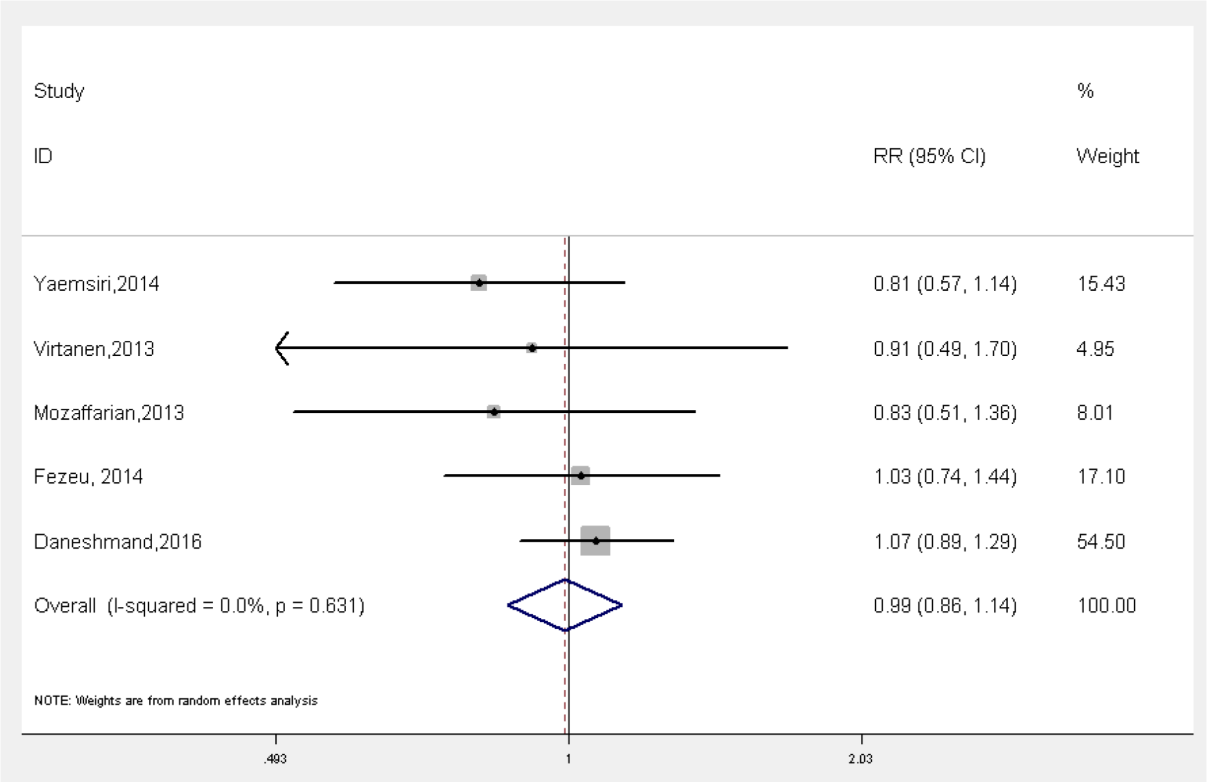

Supplementary Figure 11: Two-stage dose-response association of each 1% increment of 20:5n-3 in circulating blood with risk of stroke.
