# Supplementary material for: Circulating long-chain n-3 polyunsaturated fatty acid and incidence of stroke: a meta-analysis of prospective cohort studies
Source: Oncotarget. 2017 Jul 25;8(48):83781–91. doi: 10.18632/oncotarget.19530 (PMC5663554; doi:10.18632/oncotarget.19530)
Supplement: Supplementary file 2 [file oncotarget-08-83781-s002.docx]

| **Supplementary Tables 1. MOOSE Checklist of Present Meta-Analysis** | | |
| --- | --- | --- |
| Criteria | Comments of how the criteria were handled in the meta-analysis | Reported on page # |
|  | Reporting of background should include |  |
| Problem definition | Stroke is the second leading cause of death worldwide, and the leading cause of acquired disability in adults in most regions. Dietary n-3 PUFAs as a potentially dietary factor is closely associated with stroke risk. Most observational studies of fish or n-3 PUFA have assessed dietary intake by using food frequency questionnaires (FFQs) or weighed food records, which may have led to dietary measurement errors or bias. Circulating biomarkers of long-chain (LC) n-3 polyunsaturated fatty acid (PUFA) provide objective measures that reﬂect both dietary consumption and relevant biologic processes. Therefore, the potential public health impact of circulating levels o LC n-3 remains to be summarized quantitatively. | 2 |
| Hypothesis statement | Circulating biomarker of LC n-3 was inversely associated with incident stroke. | 3 |
| Description of study outcomes | Nevertheless, prospective cohort studies of circulating blood proportions of LC n-3 PUFA as biomarker in relation to primary prevention of stroke have yielded inconsistent results, and the optimal LC n-3 PUFA levels in circulating blood has not yet been well defined. | 3 |
| Type of exposure or intervention used | Circulating blood proportions of LC n-3 PUFA | 3 |
| Type of study designs used | Systematic review and meta-analysis. | 3 |
| Study population | Adults of any age across different countries | 4 |
|  | Reporting of search strategy should include |  |
| Qualifications of searchers(eg. librarians and investigators) | Two trained reviewers are indicated in the author list. Discrepancies unsolved by discussion during the course of study identification consulted to a third reviewer. | 4 |
| Search strategy, including time period included in the synthesis and keywords | Using a method of the key works combined with medical subject headings, and the full details were presented in supplementary data (Supplementary methods). | 4 |
| Databases and registries searched | PubMed, EMBASE and Cochrane Library database were searched, and we also check the reference lists to identify studies that might have been missed. | 4 |
| Search software used, name and version, including special features | We did not employ search software. EndNote was used to merge retrieved citations and eliminate duplications | 4, 5 |
| Use of hand searching | We hand-searched bibliographies of retrieved papers, and check the reference lists from systematic review to identify studies that might have been missed. | 4 |
| List of citations located and those excluded, including justifications | The all steps and details of the literature search process are outlined in the flow chart (Figure 1; Sup.Table 1). | 7 |
| Method of addressing articles published in languages other than English | Our search was restricted to human studies, and studies published in English. | 4 |
| Method of handling abstracts and unpublished studies | Abstract, unpublished studies and duplicated study were excluded | 4 |
| Description of any contact with authors | We did not contact authors for the detailed information of primary studies and unpublished studies. | 4 |
|  | Reporting of methods should include |  |
| Description of relevance or appropriateness of studies assembled for assessing the hypothesis to be tested | Detailed inclusion criteria were described in the methods section. | 4 |
| Rationale for the selection and coding of data | Data extracted from each of the studies were relevant to the population characteristics, study design, exposure, outcome, and adjusted confounding factors as covariates. | 5 |
| Assessment of confounding | Restricted the analysis to multiple covariates adjusted estimates. To provide a consistent approach to meta-analysis, the RR was transformed to involve comparisons between the top and the bottom tertiles of the population baseline proportions of LC n-3 PUFA in biospecimens. A sensitivity analysis was conducted by eliminating included studies one by one. Publication bias was quantitatively examined by Begg’s test and Egger’s regression test.. | 5, 7 |
| Assessment of study quality, including blinding of quality assessors; stratification or regression on possible predictors of study results | We valuated study quality and risk of bias by using the Newcastle-Ottawa scale. Subgroup analyses and meta-regression analyses were conducted to identify the sources of heterogeneity by study design, different regions, gender, baseline age, follow-up duration, stroke subtypes and biospecimen types | 6, 7 |
| Assessment of heterogeneity | Heterogeneity of the studies were explored within two types of study designs using Cochrane’s Q test of heterogeneity and I2 statistic that provides the relative amount of variance of the summary effect due to the between-study heterogeneity. | 6 |
| Description of statistical methods in sufficient detail to be replicated | Description of methods of meta-analyses for the top tertiles compared with the bottom, dose-response meta-analysis, subgroup analysis, sensitivity analyses and assessment of publication bias are detailed in the methods. | 6, 7 |
| Provision of appropriate tables and graphics | We provided 1 table and 4 figure in paper |  |
|  | Reporting of results should include |  |
| Graph summarizing individual study estimates and overall estimate | See meta-analysis for the top tertiles vs. the bottom (Figure 2 & 3) and dose-response trend (Figure 4)  All details in Sup. Figure 1-14. | 9, 10 |
| Table giving descriptive information for each study included | See characteristics of the included studies (Table 1) | 7, 8 |
| Results of sensitivity testing | See results of sensitivity analysis and subgroup analysis. (Sup. Figure S9; Table 2) | 9, 10 |
| Indication of statistical uncertainty of findings | 95% confidence intervals were presented with all summary estimates, I2 values, results of sensitivity analyses and publication analysis.( Sup. Figure S1-14) | 10 |
|  | Reporting of discussion should include |  |
| Quantitative assessment of bias | Q test and I2 statistic indicated no heterogeneity in strengths of the relationship. Evaluation of results form stratified analyses. | 12, 13 &14 |
| Justification for exclusion | We performed sensitivity analysis omitting the study to reduce the influence of potential selective bias on the overall estimate, in view of probable selection bias. | 14 |
| Assessment of quality of included studies | We discussed the results of the sensitivity analyses, and potential reasons for the observed heterogeneity. | 13 |
|  | Reporting of conclusions should include |  |
| Consideration of alternative explanations for observed results | We found that 22:6-3, but not 20:5-3, was significantly associated with lower risk of stroke. | 13 |
| Generalization of the conclusions | The present meta-analysis highlights the importance of circulating levels of LC n-3 PUFA in the development of particular subtypes of ischemic stroke. | 15 |
| Guidelines for future research | Additional well-designed observational studies and randomized clinical trials are need to evaluate the effects of individual LC n-3 PUFA on incident stroke and its specific subtypes. | 15 |
| Disclosure of funding source | See acknowledgement | 15 |
